# Acrylamide Contents of Local Snacks in Singapore

**DOI:** 10.3389/fnut.2021.764284

**Published:** 2021-12-23

**Authors:** Michelle Ting Yun Yeo, Xinyan Bi, Christiani Jeyakumar Henry

**Affiliations:** ^1^Clinical Nutrition Research Centre (CNRC), Singapore Institute of Food and Biotechnology Innovation (SIFBI), Agency for Science, Technology and Research (A^*^STAR), Singapore, Singapore; ^2^Department of Biochemistry, Yong Loo Lin School of Medicine, National University of Singapore, Singapore, Singapore

**Keywords:** acrylamide, snacks, LC-MS/MS, Singapore, Asia

## Abstract

Acrylamide is a carcinogen that forms in foods processed at high temperatures. In this study, acrylamide contents of 30 local snacks commonly consumed by the three ethnic groups (Malay, Chinese, and Indian) in Singapore were analysed by using liquid chromatography-tandem mass spectrometry (LC-MS/MS). These snacks were chosen because they were consumed regularly by people of different classes and age groups in Singapore. Our results showed that the average content of acrylamide in Indian snacks (102.23 ng/g) was higher than those in Malay (75.14 ng/g) and Chinese snacks (70.78 ng/g). The high acrylamide levels in several snacks was probably due to the processing methods and the usage of acrylamide-inducing raw materials. Same snacks prepared by different manufacturers contained different levels of acrylamide, suggesting the possibility of acrylamide reduction in these snacks. This study provides an insight into the acrylamide levels of snacks commonly consumed by the three different ethnic groups in Singapore.

## Introduction

Acrylamide is a chemical compound that has existed for as long as humans have started to utilize heat to cook food. The main pathway for acrylamide formation is the Maillard reaction, which is made up of a series of complex reactions. Formation of acrylamide begins with a condensation reaction between reducing sugars and amino acids (mainly asparagine) (1, 2). Hence, acrylamide is usually found in carbohydrate-rich foods, such as biscuits, potato chips, and French fries, dry heated at temperatures above 120°C (3–5). It has been reported that fried potato, a common snack consumed in China, contained a maximum acrylamide level of 4,126.26 μg/kg (6). Gas chromatography-mass spectrometry (GC-MS) and liquid chromatography-mass spectrometry (LC-MS) are two common methods for acrylamide analysis (7, 8). LC-MS methods are deemed to be more efficient and are easier to carry out. LC-MS acrylamide analysis methods are usually carried out using a triple quadrapole mass spectrometer and applying electrospray ionization (ESI) (3). In 2002, researchers from Swedish National Food Administration and University of Stockholm discovered the occurrence of acrylamide in food and this sparked an interest in the scientific community to research on various topics about acrylamide in food. Many factors, including the starch-containing ingredients, cooking times, cooking temperature, and even the type of leavening agent, have been reported to affect the amount of acrylamide in a given food. Therefore, many studies have been conducted to investigate the mechanism of formation of acrylamide and ways to reduce acrylamide in food (1–3, 9).

Acrylamide have considerable neurotoxic effects on humans and this have been shown by various studies (2, 10, 11). For example, one study investigated the health effects of tunnel workers exposed to a chemical grouting agent containing acrylamide (12). It was revealed that exposure to the grouting agent did result in workers having peripheral nerve system symptoms. Acrylamide is classified as a probable human carcinogen by the International Agency for Research on Cancer (IARC). Studies showed that dietary acrylamide intake is associated with esophageal cancer risk and lymphatic malignancies risk (13, 14). Apart from neurotoxic effects, the reproductive toxicity, hepatotoxicity and immunotoxicity effects have been investigated and reported in animals as well (2, 10, 11). Hence, it is of utmost importance to determine the level of acrylamide in food.

Snacking, defined as the act of eating at other times besides the main meal times, is increasingly becoming a major part of our diet. People across all age groups in Europe and United States of America are snacking at least once a day (15). A cross-sectional study, which looked at the eating behaviors of American adults, reported that the percentage of energy intake from snack consumption between lunch and dinner or snack consumption that displaced meals increased (16). In Singapore, a similar trend is also observed where the percentage of Singaporean adult residents that snacked twice or more per week increased from 18.9% in 2004 to 27.1% in 2010 (17). It was also reported that Singaporeans were consuming more sugar from food like confectionery and desserts (18).

Existing cross sectional surveys conducted in Singapore suggest that ethnicity might be a factor affecting eating habits and health in individuals (17, 18). Furthermore, Singapore is a multi-ethnic country and each of the three main ethnic groups (Malay, Chinese, and Indian) in Singapore consume different types of snacks (19–24). Therefore, it would be necessary to explore the different snacks consumed by different ethnic groups in Singapore.

Snacks are playing an increasingly major role in our diet. However, there is limited research on the level of acrylamide in local Singapore snacks. Hence, the objective of this study is to determine the acrylamide level in local snacks commonly consumed by the three major ethnic communities in Singapore.

## Materials and Methods

### Snack Samples

In total, 30 local snacks (of 3 samples each) were analysed for acrylamide content. Ten snacks representative of each ethnic group were purchased. There were three replicates for each snack and each replicate was of a different brand or was purchased from a different location or shop. All samples were homogenized using a Waring blender (Waring BB250S, Waring Commercial, CT, USA) and stored at −20°C before further analysis.

### Reagents and Standard Solutions

Stock solutions of acrylamide and labelled acrylamide (both 1.0 mg/mL in deionized water with 0.1% formic acid) were purchased from Scientific Resources (Analisa Resources, Malaysia). Both solutions were diluted with acetonitrile (Optima™ LC/MS Grade, Fisher Scientific, Waltham, MA, USA) to prepare calibration standards in the range of 0 to 1,000 ng/mL. Labelled acrylamide was diluted with 0.1% formic acid to prepare a standard solution of 5 μg/mL. For the sample cleanup procedure and LC-MS/MS analysis, hexane (Merck, KGaA, Darmstadt, Germany), anhydrous magnesium sulfate (Merck, KGaA, Darmstadt, Germany), sodium chloride (Merck, KGaA, Darmstadt, Germany), primary secondary amine (PSA) (Merck, KGaA, Darmstadt, Germany), acetonitrile (Optima™ LC/MS Grade, Fisher Scientific, Waltham, MA, USA), methanol (Optima™ LC/MS Grade, Fisher Scientific, Waltham, MA, USA), and ultrapure water from a Milli-Q IQ 7000 water purification system (Merck, KGaA, Darmstadt, Germany) were used.

### Sample Cleanup

The sample cleanup procedure to determine acrylamide contents in snacks was based on the quick, easy, cheap, effective, rugged, and safe (QuEChERS) method (25). Homogenized snack samples (1.0 ± 0.1 g) were weighed into a 50 mL centrifuge tube and 100 μL of labelled acrylamide (5 μg/mL in 0.1% formic acid) was added. Then, 5 mL of hexane, 10 mL of ultrapure water and 10 mL of acetonitrile were added and vortexed. Next, 4 g of magnesium sulfate and 0.5 g of sodium chloride were added into the mixed sample solution. To prevent formation of crystalline agglomerates, the centrifuge tube was immediately shaken and vortexed for 1 min. The centrifuge tube was then centrifuged at 5,000 rpm for 5 min. The top (hexane) layer was removed using a glass pasteur pipette and 1 mL of the middle layer was placed into a 2 mL centrifuge tube containing 150 mg of magnesium sulfate and 50 mg of PSA. The centrifuge tube was immediately vortexed and centrifuged at 5,000 rpm for 1 min. After centrifugation, the supernatant was transferred into a 2 mL amber vial for LC-MS/MS analysis.

### Liquid Chromatography–Tandem Mass Spectrometry (LC-MS/MS) Analysis

Samples were analysed on a Shimadzu CBM-20A HPLC system (Shimadzu Corporation, Kyoto, Japan) that consists of a degasser, quaternary pump, autosampler, and temperature controlled oven coupled to a Shimadzu LCMS-8050 triple quadrupole MS detector (Shimadzu Corporation, Kyoto, Japan). Separation of acrylamide was carried out on a Synergi Hydro-RP column (150 mm × 3 mm, 4 μm particle size) with a SecurityGuard Catridges C18 guard column (4 mm × 2 mm i.d.) (Phenomenex Inc., California, USA). The mobile phase was ultrapure water and methanol (99.5:0.5, v/v) at a flow rate of 0.2 mL/min. The oven temperature was 40°C and the injection volume was 10 μL. The Electrospray Ionization (ESI) source was operated in positive mode with an interface temperature of 300°C, desolvation line temperature of 250°C, and heat block temperature of 400°C. The nebulizing gas flow was 1 L/min, whereas the gas flow for heating and drying was 10 L/min. The transitions monitored for acrylamide was 72 > 55 and 72 > 27.1 while the transitions monitored for labelled acrylamide was 75 > 58.1 and 75 > 30.1. The dwell time for each transition was 247 ms. All samples and spiked samples were injected in duplicates. The calibration curve was constructed by plotting the response ratio (peak area for acrylamide: 72 > 55/peak area for labelled acrylamide: 75 > 58.1) against acrylamide concentration.

### Linearity, Recovery, and Sensitivity of Method

Prior to analysis, the method for acrylamide analysis was tested with respect to linearity, recovery and sensitivity. The method showed good linearity over a range of 0–1,000 ng/mL and the correlation coefficients for all the standard curves were >0.98 (data not shown). Three snack samples (one from each ethnic group) were spiked with different concentrations of acrylamide in duplicates (Table 1). Table 1 shows that the recoveries of the spiked samples ranged from 92.2 to 101.3%. The limit of detection (LOD) and the limit of quantification (LOQ) were determined by injecting five different low concentrations of acrylamide standards (0.1, 0.2, 0.3, 0.4, and 0.5 ng/mL) in triplicates. The LOD was evaluated to be 0.14 ng/mL based on a signal/noise ratio of 3:1 while the LOQ was evaluated to be 0.35 ng/mL based on a signal/noise ratio of 10:1.

**Table 1:** Spiking level (ng/g) and average recoveries (%) of spiked samples.

**Sample**	**Spiking level (ng/g)**	**Average recovery (%)**
Kueh Dadar _3 Replicate 1	400	99.8
Kueh Dadar_3 Replicate 2		101.3
Ear Biscuit_1 Replicate 1	400	93.6
Ear Biscuit_1 Replicate 2		99.7
Peanut Balls_3 Replicate 1	350	92.2
Peanut Balls_3 Replicate 2		96.2

### Data Analysis

Results were analysed using single factor ANOVA to determine whether there were significant differences among different ethnic groups. Single factor ANOVA was also performed to determine whether there were significant differences between different preparation methods.

## Results

**Figure 1:**
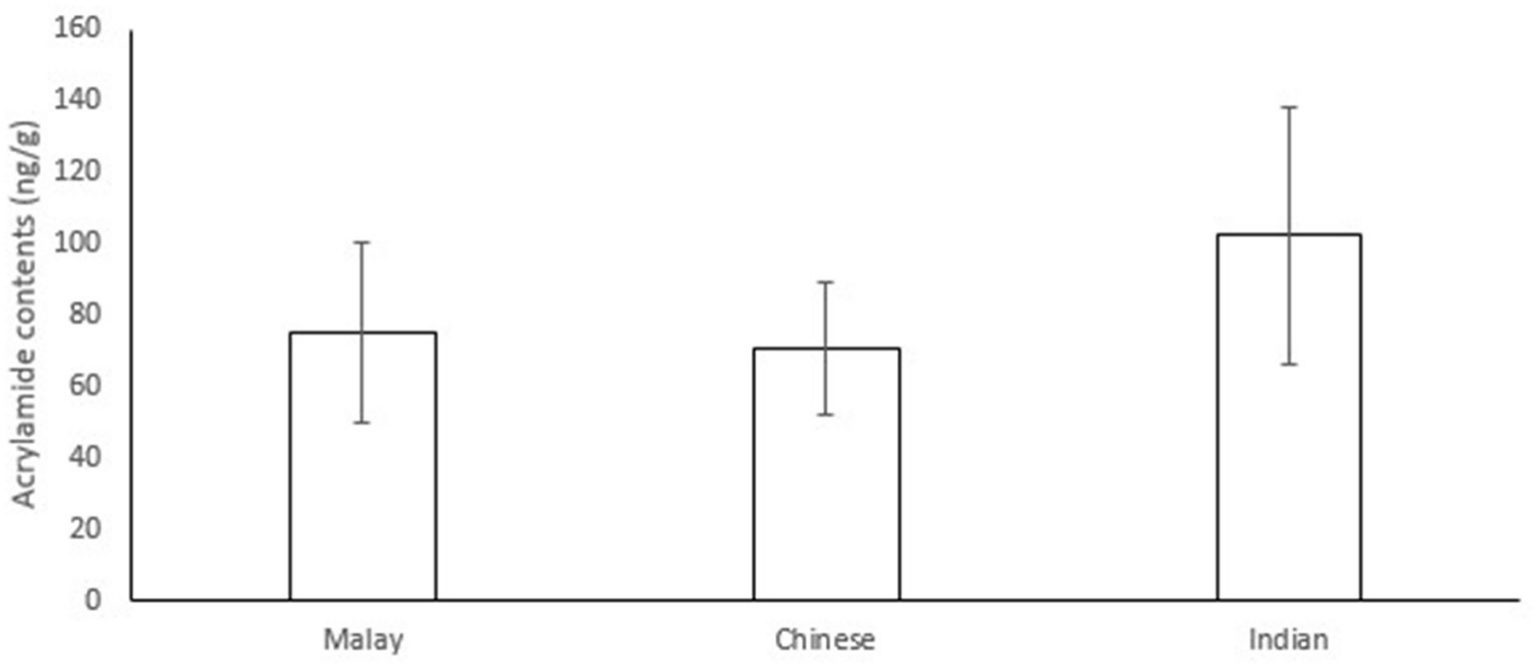
Average acrylamide contents (ng/g) ± standard errors in snacks consumed by three ethnic groups.

In the present study, acrylamide contents of 30 snacks (of 3 samples each) which are commonly consumed by three ethnic groups, i.e., Malay, Chinese, and Indian, in Singapore were analysed. The detailed information of the snacks can be found in Supplementary Figure 1 and Supplementary Table 1. Figure 1 shows the average acrylamide levels in the 30 snacks (of 3 samples each) consumed by the three ethnic groups. The average level of acrylamide in Indian snacks (102.23 ng/g) was higher than that in Malay snacks (75.14 ng/g) and Chinese snacks (70.78 ng/g).

Table 2 shows the acrylamide levels and preparation methods of 10 commonly consumed Malay snacks. Among them, Odeh Odeh_3 contained the highest amount of acrylamide (679.69 ± 15.42 ng/g) while Kueh Salat_2 was acrylamide-free. Table 3 shows the acrylamide levels and preparation methods of 10 commonly consumed Chinese snacks. Ang Ku Kueh_3 had the lowest level of acrylamide (2.59 ± 0.42 ng/g) while Ear Biscuit_1 had the highest level of acrylamide (402.66 ± 7.68 ng/g). Table 4 shows the acrylamide levels and preparation methods of 10 commonly consumed Indian snacks. Gulab Jamun_3 had the lowest level of acrylamide (1.93 ± 0.09 ng/g) while Peanut Balls_1 had the highest amount of acrylamide (1039.41 ± 19.98 ng/g).

**Table 2:** Acrylamide levels (ng/g) in commonly consumed local Malay snacks with three replicates.

**Snacks**	**Acrylamide level (ng/g)**	**Preparation method**
Kuih Bingka Ubi_1*	4.69 ± 0.00	Baked
Kuih Bingka Ubi_2*	7.66 ± 0.64	Baked
Kuih Bingka Ubi_3*	4.40 ± 0.46	Baked
Harum Manis_1	44.02 ± 4.07	Steamed
Harum Manis_2	49.39 ± 2.14	Steamed
Harum Manis_3	15.31 ± 0.16	Steamed
Curry Puff_1	105.28 ± 1.31	Deep-fried
Curry Puff_2	15.48 ± 2.09	Deep-fried
Curry Puff_3	113.71 ± 1.83	Deep-fried
Jemput Jemput Pisang_1	12.56 ± 0.37	Deep-fried
Jemput Jemput Pisang_2	21.16 ± 3.41	Deep-fried
Jemput Jemput Pisang_3	95.89 ± 5.40	Deep-fried
Kueh bahulu_1	7.66 ± 1.41	Baked
Kueh bahulu_2	7.87 ± 0.94	Baked
Kueh bahulu_3	10.81 ± 2.28	Baked
Kueh Dadar_1	16.82 ± 0.82	Pan-fried
Kueh Dadar_2	13.69 ± 0.25	Pan-fried
Kueh Dadar_3	389.00 ± 18.47	Pan-fried
Kueh Salat_1	5.53 ± 1.06	Steamed
Kueh Salat_2	N.D.	Steamed
Kueh Salat_3	4.50 ± 0.49	Steamed
Odeh Odeh_1	117.40 ± 4.92	Boiled
Odeh Odeh_2	54.66 ± 0.69	Boiled
Odeh Odeh_3	679.69 ± 15.42	Boiled
Rempeyek_1	128.53 ± 7.91	Deep-fried
Rempeyek_2	204.08 ± 7.31	Deep-fried
Rempeyek_3	95.87 ± 2.46	Deep-fried
Ongol Ubi_1	19.57 ± 2.77	Steamed
Ongol Ubi_2	5.76 ± 0.76	Steamed
Ongol Ubi_3	3.27 ± 0.18	Steamed

*Values are expressed as mean ± SD*.

* *1,2,3 are purchased from 3 different manufacturers. N.D. refers to not detected*.

**Table 3:** Acrylamide levels (ng/g) in commonly consumed local Chinese snacks with three replicates.

**Snacks**	**Acrylamide level (ng/g)**	**Preparation method**
Ang ku kueh_1*	3.14 ± 0.35	Steamed
Ang ku kueh_2*	14.59 ± 1.85	Steamed
Ang ku kueh_3*	2.59 ± 0.42	Steamed
Butterfly Fried Dough Pastry_1	68.16 ± 0.71	Deep-fried
Butterfly Fried Dough Pastry_2	20.98 ± 0.20	Deep-fried
Butterfly Fried Dough Pastry_3	50.60 ± 0.12	Deep-fried
Ear Biscuit_1	402.66 ± 7.68	Deep-fried
Ear Biscuit_2	63.96 ± 3.82	Deep-fried
Ear Biscuit_3	385.58 ± 3.92	Deep-fried
Gem Biscuit_1	200.14 ± 3.17	Baked
Gem Biscuit_2	106.11 ± 4.95	Baked
Gem Biscuit_3	108.96 ± 1.09	Baked
Heong Piah_1	167.57 ± 2.71	Baked
Heong Piah_2	61.00 ± 3.02	Baked
Heong Piah_3	118.08 ± 1.83	Baked
Red Bean Fried Dough Pastry_1	24.26 ± 0.81	Deep-fried
Red Bean Fried Dough Pastry_2	33.83 ± 0.79	Deep-fried
Red Bean Fried Dough Pastry_3	51.51 ± 0.74	Deep-fried
Salty Green Bean Pastry_1	13.42 ± 0.40	Baked
Salty Green Bean Pastry_2	29.46 ± 0.78	Baked
Salty Green Bean Pastry_3	37.50 ± 0.91	Baked
Soon Kueh_1	14.01 ± 0.39	Steamed
Soon Kueh_2	11.19 ± 0.73	Steamed
Soon Kueh_3	24.18 ± 2.45	Steamed
Wheel crackers_1	9.35 ± 1.54	Deep-fried
Wheel crackers_2	9.05 ± 0.68	Deep-fried
Wheel crackers_3	5.85 ± 1.04	Deep-fried
Wife Pastry_1	30.32 ± 2.16	Baked
Wife Pastry_2	29.67 ± 0.37	Baked
Wife Pastry_3	25.80 ± 0.32	Baked

*Values are expressed as mean ± SD*.

* *1,2,3 are purchased from 3 different manufacturers*.

**Table 4:** Acrylamide levels (ng/g) in commonly consumed local Indian snacks with three replicates.

**Snacks**	**Acrylamide level (ng/g)**	**Preparation method**
Boondi_1*	24.84 ± 0.98	Deep-fried
Boondi_2*	16.61 ± 0.09	Deep-fried
Boondi_3*	46.73 ± 0.52	Deep-fried
Gulab Jamun_1	3.77 ± 0.27	Deep-fried
Gulab Jamun_2	6.43 ± 0.34	Deep-fried
Gulab Jamun_3	1.93 ± 0.09	Deep-fried
Laddu_1	7.46 ± 0.66	Deep-fried
Laddu_2	4.80 ± 0.24	Deep-fried
Laddu_3	3.43 ± 0.26	Deep-fried
Masala Vadai_1	57.95 ± 0.87	Deep-fried
Masala Vadai_2	58.11 ± 1.85	Deep-fried
Masala Vadai_3	28.23 ± 0.71	Deep-fried
Medu Vadai_1	30.40 ± 0.23	Deep-fried
Medu Vadai_2	26.56 ± 0.47	Deep-fried
Medu Vadai_3	25.00 ± 1.50	Deep-fried
Murukku mixture_1	69.85 ± 1.63	Deep-fried
Murukku mixture_2	282.81 ± 2.08	Deep-fried
Murukku mixture_3	14.24 ± 3.38	Deep-fried
Murukku_1	124.14 ± 2.41	Deep-fried
Murukku_2	15.49 ± 2.09	Deep-fried
Murukku_3	183.06 ± 1.59	Deep-fried
Pakoda_1	82.20 ± 6.41	Deep-fried
Pakoda_2	56.63 ± 0.75	Deep-fried
Pakoda_3	126.85 ± 8.90	Deep-fried
Peanut Balls_1	1039.41 ± 19.98	Heated, roasted
Peanut Balls_2	275.34 ± 0.68	Heated, roasted
Peanut Balls_3	356.59 ± 12.14	Heated, roasted
Roasted Chickpeas_1	30.94 ± 0.78	Roasted
Roasted Chickpeas_2	38.31 ± 0.37	Roasted
Roasted Chickpeas_3	28.86 ± 1.68	Roasted

*Values are expressed as mean ± SD*.

* *1,2,3 are purchased from 3 different manufacturers*.

With reference to the single factor ANOVA analysis, there was no significant difference between the three ethnic groups in terms of the acrylamide contents of snacks (*p*-value of 0.687 > 0.05). However, the different preparation methods of snacks have a significant impact on the acrylamide contents of snacks (*p*-value of 4.15 × 10^−9^ <0.05). Specifically, the following pair of preparation methods have a significant impact on the acrylamide contents of snacks: baked and steamed; baked and boiled; baked and heated, roasted; steamed and boiled, steamed and deep-fried, steamed and pan-fried; steamed and heated, roasted; boiled and deep fried; deep-fried and heated, roasted.

## Discussion

Acrylamide is found in many food sources, especially deep-fried or baked snacks (26). As more and more Singaporeans consumed sweet desserts and snacks (17, 18), it is of utmost importance to know the acrylamide contents in the commonly consumed snacks by Singaporeans. The Food and Agriculture Organization/World Health Organization (FAO/WHO) estimated the average acrylamide intake for the general population to be around 0.3 to 0.8 μg per kilogram of bodyweight per day (27). Therefore, the tolerable daily intake (TDI) is 21–56 μg for a 70 kg human.

In this study, 30 commonly consumed snacks (of 3 samples each) were analysed for acrylamide content. Among these snacks, 8 snacks, including 2 Malay snacks, Kueh Dadar_3 and Odeh Odeh_3, 2 Chinese snacks, Ear Biscuit_1 and Ear Biscuit_3, and 4 Indian snacks, Murukku Mixture_2, Peanut Balls_1, Peanut Balls_2, and Peanut Balls_3, had acrylamide levels more than 210 ng/g, which exceeded TDI if 100 g of the snacks were consumed.

The average level of acrylamide in Indian snacks was higher than that in Malay and Chinese snacks. This could be attributed to their preparation method. For Indian snacks, they were prepared by using deep-fried or roasting methods. Murukku Mixture and Murukku, two common Indian snacks, are usually prepared by frying the rice flour mixture in hot oil heated up to 180°C (28). Therefore, both snacks contained high levels of acrylamide due to heating starch-containing ingredients at high temperatures. Ear Biscuit, a Chinese snack, and Rempeyek, a Malay snack, which are prepared by deep-frying, had higher levels of acrylamide. Our results are consistent with previous studies showing that deep-fried food had a higher amount of acrylamide (29). Compared to baked goods, fried goods largely contained a higher level of acrylamide (30). Michalak et al. (31) reported that deep-fried croquettes generally had higher acrylamide content compared to pan-fried croquettes. Other studies showed that the level of acrylamide in fried goods and baked goods such as biscuits were generally higher compared to other food products (6, 32).

Among the 10 snacks, the Peanut Balls, one of the Indian snacks, purchased from 3 different shops contain a very high level of acrylamide with a maximum value of 1,039.41 ng/g. The high levels of acrylamide present in Peanut Balls are probably due to the following factors. Peanut Balls are made by pouring hot jaggery, which is sugarcane syrup, over the roasted peanuts (33). The high temperature of roasting peanuts and heating jaggery could contribute to the high acrylamide levels in Peanut Balls. On the other hand, Phaeon et al. (34) recently reported that a maximum amount of acrylamide (4,011 ng/g) was found in jaggery. Moreover, the main amino acid present in jaggery is asparagine, which reacts with reducing sugars to form acrylamide (34). The main carbohydrate in jaggery is sucrose (35). The decomposition of sucrose under high heat might have contributed to the high acrylamide content in Peanut Balls in two ways. Firstly, it produces neo-carbonyls that facilitates acrylamide formation (36). Secondly, it produces reducing sugars that could participate in the Maillard reaction (37). Although peanuts are not high in asparagine, it contains other amino acids that could be precursors for the formation of acrylamide (38).

Kueh Dadar and Odeh Odeh are two commonly consumed Malay snacks. The main ingredients used in making Kueh Dadar is coconut milk and palm sugar syrup, while for Odeh Odeh, one of the main ingredients used is palm sugar syrup too. Therefore, the presence of palm sugar syrup in Kueh Dadar and Odeh Odeh may explain the high levels of acrylamide. Like jaggery, palm sugar syrup contains acrylamide with a maximum content of 225 ng/g (34). Similarly, the main amino acid in palm sugar syrup is asparagine (34). Additionally, for Kueh Dadar, it uses coconut milk to prepare. The main amino acid in coconut milk is glutamic acid (39). It has been reported that glutamic acid could be a possible acrylamide precursor because during heating of coconut milk, it was observed that only glutamic acid decreased as the amount of acrylamide increased (40).

Same snacks prepared by different manufacturers contained different acrylamide levels (41). This is probably due to several factors such as the different types of raw material used or the different heating temperature and time applied during the cooking process. Our study also shows that the acrylamide contents of snacks was significantly influenced by different preparation methods. Although it is not possible to eliminate acrylamide completely from food, we can reduce acrylamide levels by choosing appropriate raw materials, adding additives, and altering processing conditions. The level of reducing sugars and asparagine in the raw materials have a major impact on the resulting acrylamide level in food. The content of these two compounds in raw materials vary with the climate and storage conditions, fertilization techniques and the manufacturing processes (2, 42). For example, the level of asparagine in cane syrup was slightly different when purchased from a different province (34). Choosing raw materials with lower content of reducing sugars and asparagine would lower the acrylamide content in food.

The addition of additives like asparaginase, lysine, glycine, antioxidants, vitamins and salt solutions are known to reduce acrylamide formation (1, 2). It was reported that the addition of asparaginase into whole wheat flour reduced the formation of acrylamide in the resulting bread crisp by 88% (43). The addition of glycine into whole wheat flour also reduced the formation of acrylamide in the resulting bread crisp by more than 30% (43). Urbančič et al. (44) investigated the effect of adding rosemary extract, a natural antioxidant, to sunflower oil before frying of potato fries and it was observed that the acrylamide level in potato fries decreased by 37%.

The reduction in acrylamide formation can also be achieved by changing the processing conditions such as heating temperature and time, pH, and water content of food (1, 2). Increased frying temperature and time lead to an increase in acrylamide content in potato fries (9). A similar trend was also observed in baked fries, where the acrylamide content increased with baking temperature and time (45). Hence, heating temperature and time could be controlled to decrease acrylamide content in food. Other processing conditions like the type of frying oil used for deep-frying and the number of frying cycles influences the level of acrylamide in food (46). This could explain why the same snacks prepared by different manufacturers like Murukku, contained different acrylamide levels. In general, boiled or steamed foods contain traces of acrylamide. However, in this study, snacks such as Odeh odeh, which is prepared by boiling, contained a maximum acrylamide level of 679.69 ng/g. A decrease in pH of foods can reduce acrylamide by preventing the formation of the Schiff base, which is an intermediate compound to form acrylamide (47). Previous studies have shown that lower acrylamide contents existed in bakery products with lower pH (4). Moreover, the addition of citric acid before heating potatoes in an oven decreased acrylamide formation in the potatoes (45).

Other methods, i.e., using sugarcane syrup with a lower level of asparagine to prepare Peanut Balls, could be applied to reduce the acrylamide levels. Asparaginase could also be added into flour to reduce the acrylamide content of Ear biscuit. Mitigation strategies for acrylamide and the effects of these strategies on the acrylamide levels of the snacks could be explored in future studies.

## Conclusion

Thirty local snacks (of 3 samples each) commonly consumed by the three ethnic groups in Singapore were analysed. The acrylamide levels for Malay, Chinese and Indian snacks ranged between not detected to 679.69, 2.59–402.66 and 1.93–1,039.41 ng/g, respectively. In general, the acrylamide levels of local snacks consumed in Singapore were low. Eight snacks had an acrylamide level more than 210 ng/g, which exceeded TDI if 100 g of the snacks were consumed per day. Compared to Chinese and Malay snacks, Indian snacks had a higher average acrylamide level, probably because Indian snacks were generally prepared by using deep-fried or roasting methods. Same snacks prepared by different manufacturers contained different acrylamide levels. Therefore, it could be possible to reduce the acrylamide contents of snacks by changing the processing conditions or choosing raw materials containing lesser acrylamide. Further mitigation strategies are needed to reduce the acrylamide levels in commonly consumed local snacks and foods.

## Data Availability Statement

The original contributions presented in the study are included in the article/Supplementary Material, further inquiries can be directed to the corresponding author/s.

## Author Contributions

CH and XB: conceptualization. MY: methodology and investigation. MY and XB: validation, formal analysis, and writing—original draft preparation. MY, XB, and CH: writing—review and editing. CH: supervision and project administration. All authors contributed to the article and approved the submitted version.

## Funding

This research was funded by IAF-PP Food Structure Engineering for Nutrition and Health Programme (Grant ID Nos: H17/01/a0/A11 and H18/01/a0/B11).

## Conflict of Interest

The authors declare that the research was conducted in the absence of any commercial or financial relationships that could be construed as a potential conflict of interest.

## Publisher's Note

All claims expressed in this article are solely those of the authors and do not necessarily represent those of their affiliated organizations, or those of the publisher, the editors and the reviewers. Any product that may be evaluated in this article, or claim that may be made by its manufacturer, is not guaranteed or endorsed by the publisher.

## References

[1] Pedreschi F Mariotti MS Granby K. Current issues in dietary acrylamide: formation, mitigation and risk assessment. J Sci Food Agric. (2013) 94:9–20. 10.1002/jsfa.6349 23939985

[2] Rifai L Saleh FA. A review on acrylamide in food: occurrence, toxicity, and mitigation strategies. Int J Toxicol. (2020) 39:93–102. 10.1177/1091581820902405 32013673

[3] Lineback DR Coughlin JR Stadler RH. Acrylamide in foods: a review of the science and future considerations. Annu Rev Food Sci Technol. (2012) 3:15–35. 10.1146/annurev-food-022811-101114 22136129

[4] Sarion C Codină GG Dabija A. Acrylamide in bakery products: a review on health risks, legal regulations and strategies to reduce its formation. Int J Environ Res Public Health. (2021) 18:4332. 10.3390/ijerph18084332 33921874 PMC8073677

[5] Timmermann CAG Molck SS Kadawathagedara M Bjerregaard AA Törnqvist M Brantsæter AL. A review of dietary intake of acrylamide in humans. Toxics. (2021) 9:155. 10.3390/toxics9070155 34209352 PMC8309717

[6] Chen Y-H Xia E-Q Xu X-R Ling W-H Li S Wu S. Evaluation of Acrylamide in Food from China by a LC/MS/MS Method. Int J Environ Res Public Health. (2012) 9:4150–8. 10.3390/ijerph9114150 23202837 PMC3524618

[7] Mollakhalili-Meybodi N Khorshidian N Nematollahi A Arab M. Acrylamide in bread: a review on formation, health risk assessment, and determination by analytical techniques. Environ Sci Pollut Res. (2021) 28:15627–45. 10.1007/s11356-021-12775-3 33548042

[8] Skinner MM Seale JT Cantrell MS Collins JM Turner MW McDougal OM. Instrumentation for routine analysis of acrylamide in French Fries: assessing limitations for adoption. Foods. (2021) 10:2038. 10.3390/foods10092038 34574148 PMC8469642

[9] Vinci RM Mestdagh F Meulenaer BD. Acrylamide formation in fried potato products—Present and future, a critical review on mitigation strategies. Food Chem. (2012) 133:1138–54. 10.1016/j.foodchem.2011.08.001

[10] Zamani E Shokrzadeh M Fallah M Shaki F. A review of acrylamide toxicity and its mechanism. Pharm Biomed Res. (2017) 3:1–7. 10.18869/acadpub.pbr.3.1.1

[11] Kumar J Das S Teoh SL. Dietary acrylamide and the risks of developing cancer: facts to ponder. Front Nutr. (2018) 5:14. 10.3389/fnut.2018.00014 29541638 PMC5835509

[12] Hagmar L Törnqvist M Nordander C Rosén I Bruze M Kautiainen A. Health effects of occupational exposure to acrylamide using hemoglobin adducts asbiomarkers of internal dose. Scand J Work Environ Health. (2001). 27:219−226. 10.5271/sjweh.608 11560335

[13] Lin Y Lagergren J Lu Y. Dietary acrylamide intake and risk of esophageal cancer in a population-based case-control study in Sweden. Int J Cancer. (2011) 128:676–81. 10.1002/ijc.25608 20715108

[14] Bongers Ml Hogervorst JGF Schouten LJ Goldbohm RA Schouten HC. Dietary acrylamide intake and the risk of lymphatic malignancies: The Netherlands cohort study on diet and cancer. PLoS ONE. (2012) 7:e38016. 10.1371/journal.pone.0038016 22723843 PMC3377662

[15] Bellisle F. Meals and snacking, diet quality and energy balance. Physiol Behav. (2014) 134:38–43. 10.1016/j.physbeh.2014.03.010 24657181

[16] Kant AK Graubard BI. 40-year trends in meal and snack eating behaviors of American adults. J Acad Nutr Diet. (2015) 115:50–63. 10.1016/j.jand.2014.06.354 25088521 PMC4276433

[17] Health Promotion Board Singapore. Report of the National Nutrition Survey 2010. (2010). Singapore: Health Promotion board. https://www.hpb.gov.sg/docs/default-source/pdf/nns-2010-report.pdf?sfvrsn=18e3f172 2 (accessed on May 31, 2021).

[18] Health Promotion Board Singapore. National Nutrition Survey 2018 Shows Gradual Improvements in Singaporeans' Dietary Habits. (2018). https://www.hpb.gov.sg/article/national-nutrition-survey-2018-shows-gradual-improvements-in-singaporeans-dietary-habits (accessed on May 31, 2021).

[19] Lee S. A New Taste of Tradition: Chinese Snacks and Hawker-Entrepreneurs in Singapore. (2008) (dissertation thesis) (Curtin Theses) Curtin University.

[20] Gowri B Devi KPV. Microbial quality of food products sold by self help group women of informal sectors in Tamilnadu State, India. Indian J Sci Technol. (2012) 5:1967–9. 10.17485/ijst/2012/v5i1.34

[21] Gupta V Downs SM Ghosh-Jerath S Lock K Singh A. Unhealthy fat in street and snack foods in low-socioeconomic settings in india: a case study of the food environments of rural villages and an urban slum. J Nutr Educ Behav. (2016) 48:269–79. 10.1016/j.jneb.2015.11.006 26872553 PMC4826272

[22] Neelakantan N Whitton C Seah S Koh H Rebello SA Lim JY. Development of a semi-quantitative food frequency questionnaire to assess the dietary intake of a multi-ethnic urban Asian population. Nutrients. (2016) 8:528. 10.3390/nu8090528 27618909 PMC5037515

[23] Raji MNA Karim SA Ishak FAC Arshad MM. Past and present practices of the Malay food heritage and culture in Malaysia. J Ethn Foods. (2017) 4:221–31. 10.1016/j.jef.2017.11.001

[24] Soon S-C Xing EY Tong CK. The Singapore Ethnic Mosaic: Many Cultures, One People. (2018). Singapore: World Scientific Publishing Co. Pte. Ltd.

[25] Mastovska K Lehotay SJ. Rapid sample preparation method for LC–MS/MS or GC–MS analysis of acrylamide in various food matrices. J Agric Food Chem. (2006) 54:7001–8. 10.1021/jf061330r 16968055

[26] Maan AA Anjum MA Khan MKI Nazir A Saeed F Afzaal M. Acrylamide formation and different mitigation strategies during food processing-A review. Food Rev Int. (2020) 1–18. 10.1080/87559129.2020.1719505

[27] World Health Organization. Health Implications of Acrylamide in Food: Report of a Joint FAO/WHO Consultation, WHO Headquarters. (2002). Geneva, Switzerland, 25–27 June 2002. https://apps.who.int/iris/bitstream/handle/10665/42563/9241562188.pdf?sequence=1 (Accessed June 1, 2021).

[28] Sarangam S Chakraborty P Chandrasheker G. Development of low fat multigrain murukku—a traditional savoury product. IJRAF. (2015) 2:15–24.

[29] Palazoglu TK Savran D Gökmen V. Effect of cooking method (baking compared with frying) on acrylamide level of potato chips. J Food Sci. (2010) 75:25–9. 10.1111/j.1750-3841.2009.01389.x 20492162

[30] Wang H Feng F Guo Y Shuang S Choi MMF. HPLC-UV quantitative analysis of acrylamide in baked and deep-fried Chinese foods. J Food Compos Anal. (2013) 31:7–11. 10.1016/j.jfca.2013.02.006

[31] Michalak J Gujska E Czarnowska-Kujawska M Nowak F. Effect of different home-cooking methods on acrylamide formation in pre-prepared croquettes. J Food Compos Anal. (2017) 56:134–9. 10.1016/j.jfca.2016.12.006

[32] Senyuva HZ Gökmen V. Survey of acrylamide in Turkish foods by an in-house validated LC-MS method. Food Addit Contam. (2005) 22:204–9. 10.1080/02652030512331344178 16019788

[33] Pallavi BV Chetana R Reddy SY. Processing, physico-chemical, sensory and nutritional evaluation of protein, mineral and vitamin enriched peanut chikki—an Indian traditional sweet. J Food Sci Technol. (2014) 51:158–62. 10.1007/s13197-011-0467-0 24426063 PMC3857401

[34] Phaeon N Chapanya P Mueangmontri R Pattamasuwan A Lipan L Carbonell-Barrachina ÁA. Acrylamide in non-centrifugal sugars and syrups. J Sci Food Agric. (2021) 101:4561–9. 10.1002/jsfa.11098 33460464

[35] Singh J Solomon S Kumar D. Manufacturing jaggery, a product of sugarcane, as health food. Agrotechnology. (2013). S11:007. 10.4172/2168-9881.S11-007

[36] Kocadagli T Goncuoglu N Hamzalioglu A Gokmen V. In depth study of acrylamide formation in coffee during roasting: role of sucrose decomposition and lipid oxidation. Food Funct. (2012). 3:970–975. 10.1039/c2fo30038a 22796869

[37] Bent G-A Maragh P Dasgupta T. Acrylamide in Caribbean foods—residual levels and their relation to reducing sugar and asparagine content. Food Chem. (2012) 133:451–7. 10.1016/j.foodchem.2012.01.067 25683419

[38] Yaylayan VA Locas CP Wnorowski A O'Brien J. Mechanistic pathways of formation of acrylamide from different amino acids. In: Friedman M, Mottram D. Chemistry and Safety of Acrylamide in Food. Chemistry and Safety of Acrylamide in Food. USA: Springer. p. 191–204. 10.1007/0-387-24980-X_15 16438299

[39] Patil U Benjakul S. Coconut milk and coconut oil: their manufacture associated with protein functionality. J Food Sci. (2018) 83:2019–27. 10.1111/1750-3841.14223 30004125

[40] Jom KN Jamnong P Lertsiri S. Investigation of acrylamide in curries made from coconut milk. Food Chem Toxicol. (2007) 46:119–24. 10.1016/j.fct.2007.07.006 18029078

[41] González-Mulero L Mesías M Morales FJ Delgado-Andrade C. Acrylamide exposure from common culinary preparations in Spain, in household, catering and industrial settings. Foods. (2021) 10:2008. 10.3390/foods10092008 34574118 PMC8467121

[42] Muttucumaru N Powers SJ Elmore JS Dodson A Briddon A Mottram DS. Acrylamide-forming potential of potatoes grown at different locations, and the ratio of free asparagine to reducing sugars at which free asparagine becomes a limiting factor for acrylamide formation. Food Chem. (2017) 220:76–86. 10.1016/j.foodchem.2016.09.199 27855938 PMC5119237

[43] Capuano E Ferrigno A Acampa I Serpen A Açar ÖÇ Gökmen V. Effect of flour type on Maillard reaction and acrylamide formation during toasting of bread crisp model systems and mitigation strategies. Int Food Res. (2009) 42:1295–302. 10.1016/j.foodres.2009.03.018

[44] Urbančič S Kolar MH Dimitrijević D Demšar L Vidrih R. Stabilisation of sunflower oil and reduction of acrylamide formation of potato with rosemary extract during deep-fat frying. LWT. (2014). 57, 671–678. 10.1016/j.lwt.2013.11.002

[45] Rydberg P Eriksson S Tareke E Karlsson P Ehrenberg L Tornqvist M. Investigations of factors that influence the acrylamide content of heated foodstuffs. J Agric Food Chem. (2003) 51:7012–8. 10.1021/jf034649+ 14611163

[46] Gertz C Klostermann S Kochhar SP. Deep frying: the role of water from food being fried and acrylamide formation. OCL—Oilseeds Fats Crops Lipids. (2003) 10:297–303. 10.1051/ocl.2003.0297

[47] Mestdagh F Maertens J Cucu T Delporte K Peteghem CV Meulenaer BD. Impact of additives to lower the formation of acrylamide in a potato model system through pH reduction and other mechanisms. Food Chem. (2008) 107:26–31. 10.1016/j.foodchem.2007.07.013

