# Supplementary material for: Acrylamide Contents of Local Snacks in Singapore
Source: Front Nutr. 2021 Dec 23;8:764284. doi: 10.3389/fnut.2021.764284 (PMC8733551; doi:10.3389/fnut.2021.764284)
Supplement: Supplementary file 1 [file Image_1.pdf]

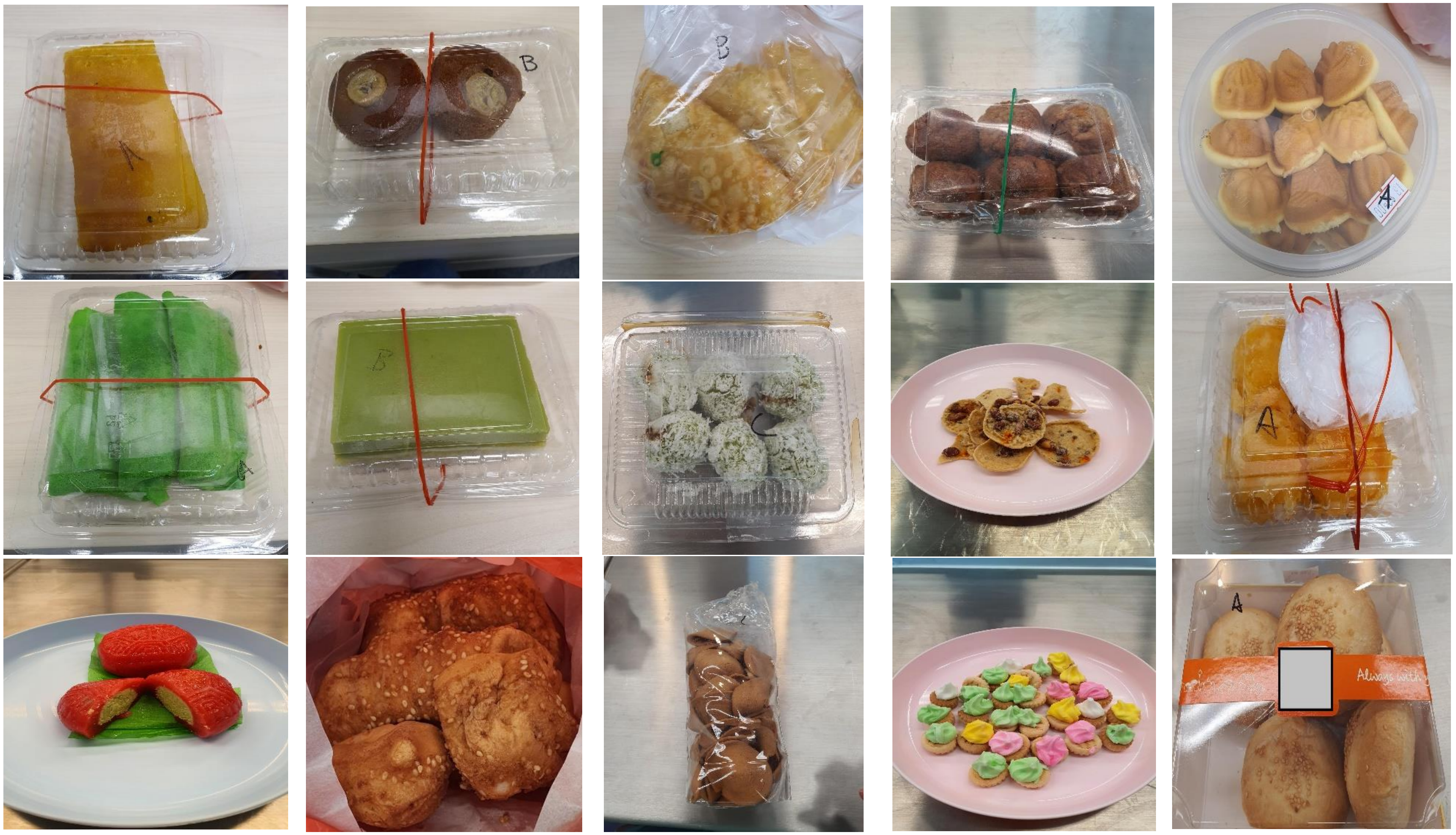
Always with

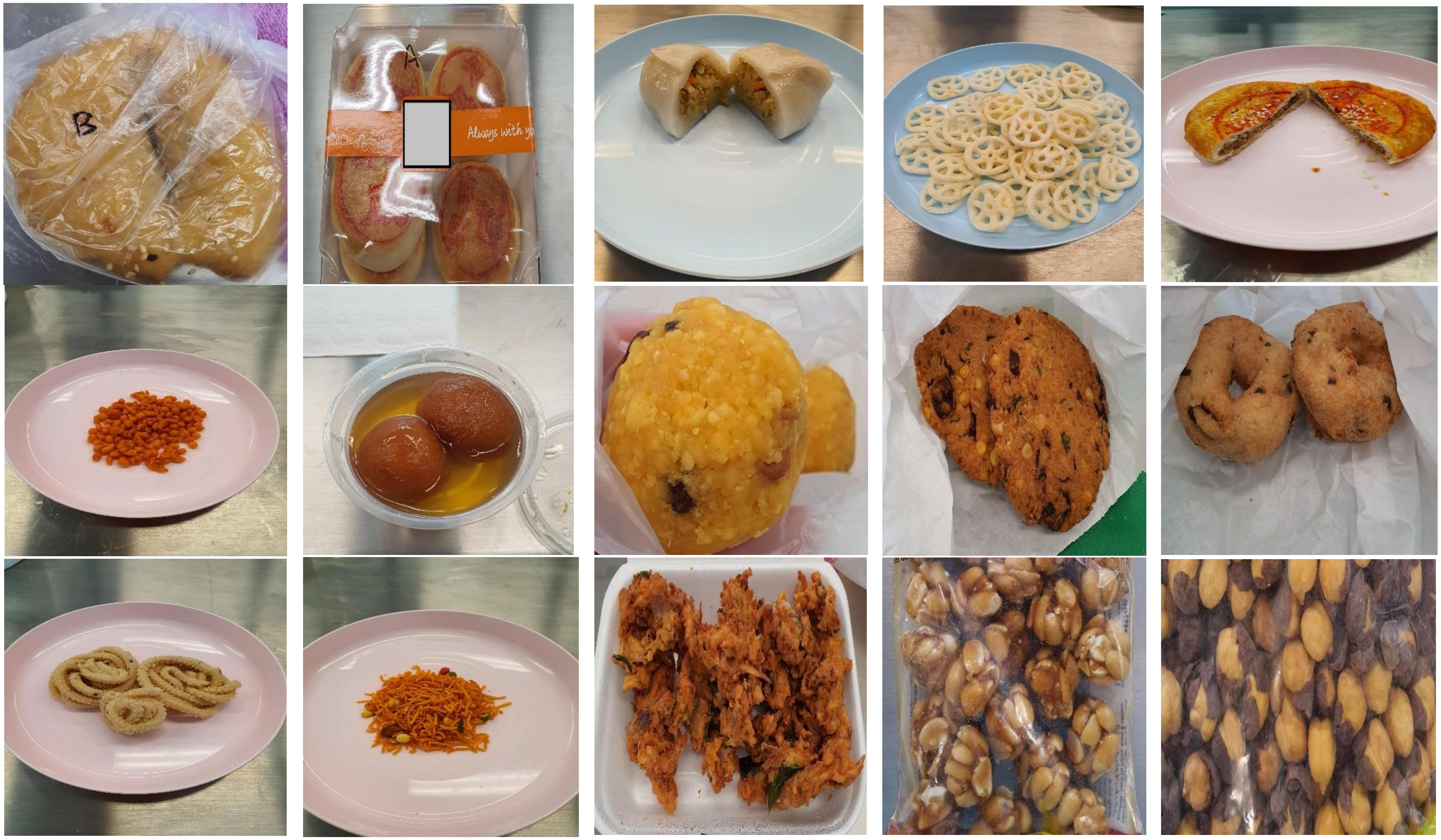
B
A
Always with

Figure S1: Local snacks investigated in the study

Row 1 (from left to right): Kuih Bingka Ubi, Harum Manis, Curry Puff, Jemput Jemput Pisang, Kueh bahulu

Row 2 (from left to right): Kueh Dadar, Kueh Salat, Odeh Odeh, Rempeyek, Ongol Ubi (white coconut shreds not added into sample)

Row 3 (from left to right): Ang ku kueh, Butterfly Fried Dough Pastry, Ear Biscuit, Gem biscuit, Heong Piah

Row 4 (from left to right): Red Bean Fried Dough Pastry, Salty Green Bean pastry, Soon Kueh, Wheel crackers, Wife Pastry

Row 5 (from left to right): Boondi, Gulab Jamun, Laddu, Masala Vadai, Medu Vadai

Row 6 (from left to right): Murukku, Murukku mixture, Pakoda, Peanut Balls, Roasted Chickpeas
