# Supplementary material for: Acrylamide Contents of Local Snacks in Singapore
Source: Front Nutr. 2021 Dec 23;8:764284. doi: 10.3389/fnut.2021.764284 (PMC8733551; doi:10.3389/fnut.2021.764284)
Supplement: Supplementary file 2 [file Table_1.docx]

Table S1. Brief Description of local snacks. *Ang ku kueh analysed in this study was glutinous rice flour skin wrapped with sweet mung bean filling.

| **Snacks** | **Description** |
| --- | --- |
| Peanut Balls | Sweet snack made from pouring hot jaggery over roasted peanuts |
| Ear Biscuit | Deep-fried ear-shaped biscuits made from wheat flour |
| Odeh Odeh | Glutinous rice flour balls filled with palm sugar syrup and coated with grated coconut |
| Rempeyek | Deep-fried cracker made from rice flour and topped with ingredients such as peanuts |
| Kueh Dadar | Rolled crepe made using wheat flour and coconut milk and filled with grated coconut cooked in palm sugar syrup |
| Gem biscuit | Biscuit made using wheat flour and topped with icing |
| Murukku mixture | Deep-fried fritters made from dhall flour and rice flour and mixed with other ingredients like peanuts and curry leaves |
| Heong Piah | Pastries with malt filling |
| Murukku | Deep-fried fritters made from dhall flour and rice flour |
| Pakoda | Deep-fried fritters made from gram flour |
| Curry Puff | Deep-fried pies filled with ingredients, often curry potatoes and chicken |
| Masala Vadai | Deep-fried fritters made using chana dal (split chickpeas) |
| Butterfly Fried Dough Pastry | Deep-fried dough fritters made from wheat flour |
| Jemput Jemput Pisang | Deep-fried banana fritters shaped like a ball |
| Red Bean Fried Dough Pastry | Deep-fried dough fritters filled with red bean paste |
| Harum Manis | Sponge cake topped with banana |
| Roasted Chickpeas | Roasted chickpeas |
| Boondi | Deep-fried snack made from gram chickpea flour |
| Wife Pastry | Pastries (made using wheat flour) with winter melon filling |
| Medu Vadai | Deep-fried fritters made using Vigna mungo, a type of bean |
| Salty Green Bean pastry | Pastries with mung bean filling |
| Soon Kueh | Steamed dumpling (made using rice flour and tapioca flour) filled with shredded bamboo shoots, turnips and dried shrimps |
| Ongol Ubi | Steamed tapioca cake |
| Kueh bahulu | Baked egg sponge cake |
| Wheel crackers | Deep-fried crackers made from wheat flour and potato starch |
| Ang ku kueh | Glutinous rice flour skin wrapped with a salty or sweet filling.* |
| Kuih Bingka Ubi | Baked tapioca cake |
| Laddu | Deep-fried snack commonly made from gram chickpea flour and sugar |
| Gulab Jamun | Deep fried snack made from milk solids or milk powder and soaked in hot syrup |
| Kueh Salat | 2 layer kueh that consists of glutinous rice topped with pandan-flavoured egg custard |
